# Trends of rubella incidence during a 5-year period of case based surveillance in Zimbabwe

**DOI:** 10.1186/s12889-015-1642-4

**Published:** 2015-03-27

**Authors:** Simbarashe Chimhuya, Portia Manangazira, Arnold Mukaratirwa, Pasipanodya Nziramasanga, Chipo Berejena, Annie Shonhai, Mary Kamupota, Regina Gerede, Mary Munyoro, Douglas Mangwanya, Christopher Tapfumaneyi, Charles Byabamazima, Eshetu Messeret Shibeshi, Kusum Jackison Nathoo

**Affiliations:** Department of Paediatrics and Child Health, University of Zimbabwe-College of Health Sciences, Mazoe Street, A178 Avondale, Harare Zimbabwe; Epidemiology and Disease Control Directorate, Ministry of Health and Child Care, Harare, Zimbabwe; Medical Microbiology Department, University of Zimbabwe-College of Health Sciences, Mazoe Street, A178 Avondale, Harare Zimbabwe; Expanded Programme of Immunization, Ministry of Health and Child Care, Harare, Zimbabwe; Expanded Programme of Immunization, World Health Organization, Harare, Zimbabwe; Laboratory Services Directorate, Ministry of Health and Child Care, Harare, Zimbabwe; Curative Services Directorate, Ministry of Health and Child Care, Harare, Zimbabwe; Immunization and Vaccines Development, East and South Africa Inter-Country Support Team, World Health Organization, Harare, Zimbabwe

**Keywords:** Rubella, Zimbabwe, Seroprevalence, Incidence, Surveillance, Trends

## Abstract

**Background:**

Rubella is a disease of public health significance owing to its adverse effects during pregnancy and on pregnancy outcomes. Women who contract rubella virus during pregnancy may experience complications such as foetal death or give birth to babies born with congenital rubella syndrome. Vaccination against rubella is the most effective and economical approach to control the disease, and to avoid the long term effects and high costs of care for children with congenital rubella syndrome as well as to prevent death from complications. Zimbabwe commenced rubella surveillance in 1999, despite lacking a rubella vaccine in the national Expanded Programme on Immunization, as per the World Health Organization recommendation to establish a surveillance system to estimate the disease burden before introduction of a rubella vaccine. The purpose of this analysis is to describe the disease trends and population demographics of rubella cases that were identified through the Zimbabwe national measles and rubella case-based surveillance system during a 5-year period between 2007 and 2011.

**Methods:**

Data from the Zimbabwe National Measles Laboratory for the 5-year study period were analysed for age, sex, district of origin, seasonality, and rubella IgM serostatus.

**Results:**

A total of 3428 serum samples from cases of suspected measles in all administrative districts of the country were received by the laboratory during this period. Cases included 51% males and 49% females. Of these, 2999 were tested for measles IgM of which 697 (23.2%) were positive. Of the 2302 measles IgM-negative samples, 865 (37.6%) were rubella IgM-positive. Ninety-eight percent of confirmed rubella cases were children younger than 15 years of age. Most infections occurred during the dry season.

**Conclusions:**

The national case-based surveillance revealed the disease burden and trends of rubella in Zimbabwe. These data add to the evidence for introducing rubella-containing vaccine into the national immunization programme.

**Electronic supplementary material:**

The online version of this article (doi:10.1186/s12889-015-1642-4) contains supplementary material, which is available to authorized users.

## Background

Rubella is a disease of public health significance, largely owing to the teratogenic effects of the virus, and is characterized by multiple birth defects known as congenital rubella syndrome (CRS). Common birth defects are ocular (cataracts, retinitis, microphthalmia, and glaucoma), hearing impairment, heart defects (pulmonary stenosis, persistent ductus arteriosus), microcephaly, developmental delay, mental retardation, bone alterations, and damage to the liver and spleen [1]. Other adverse outcomes of rubella infection in early pregnancy or just before conception include foetal resorption, spontaneous abortion and intrauterine foetal death. It is estimated that the majority (90%) of infants with CRS are born to women who were infected by rubella virus in the first 10 weeks of pregnancy [2].

It is estimated that more than 100,000 infants worldwide are born with CRS each year. Africa, the Western Pacific and Southeast Asia are regions known to have the highest burden of CRS [3,4]. The incidence of CRS is estimated to be between 0.1 and 0.2 cases per 1000 live births. This incidence rises to 1–4 cases per 1000 live births during rubella outbreaks. In the prevaccine era, rubella was responsible for over 11,000 foetal deaths and there were 20,000 infants born with CRS in the United States during an epidemic between 1964 and 1965. Routine rubella vaccination in the US began in 1969, and by 2004, the country was declared free of endemic rubella [5].

Nearly 136,000 cases of rubella were reported in the Americas in 1998, predominantly in southern regions. In 2003, Pan American Health Organization member countries established a goal to eliminate rubella and CRS from the Western Hemisphere by 2010, under a background of outbreaks during the 1990s [6]. This goal was achieved when the last cases of endemic rubella were reported in 2009, and the eight cases reported in Canada and the US were found to be imported [7].

The World Health Organization (WHO) European Region set a measles and rubella elimination goal by 2010 (resolution EUR/RC55/R7 of 2005) under a background of rising rubella incidence, especially in central and Eastern Europe and the post-Soviet states [8]. Through increased immunization efforts, the incidence of rubella was reduced from 233 cases in 2005 to 13 in 2009 per 100,000 population [9].

Meanwhile, rubella has been circulating widely in Africa. Estimates of rubella prevalence are obtained primarily from combined measles/rubella case-based surveillance conducted according to the guidelines of the WHO Regional Office for Africa (WHO/AFRO) [10]. Goodson et al. analysed rubella seroprevalence data from Africa from 17 published reports and estimated that 1–29% of adults and 6–16% of women of child-bearing age (15 to 49 years) in the region are susceptible to rubella [11]. The majority (95%) of rubella cases reported in this survey had occurred in children up to 14 years of age.

In Zimbabwe, surveillance for rubella was introduced in 1999 through the existing measles surveillance system. The measles case definition (children and adults presenting to health facilities with rash and fever plus at least one of the following: coryza, conjunctivitis or cough, or any person in whom a clinician suspects measles) was also adopted for rubella surveillance. All samples negative for measles IgM antibodies by serological testing are subsequently tested for rubella. This approach may underestimate the burden of rubella because the surveillance is not primarily designed to identify rubella but rather to identify measles. Some rubella infections are missed because they do not meet the “suspected measles” case definition. The WHO/AFRO measles surveillance guidelines require that the testing algorithm exclude measles IgM-positive cases from rubella screening.

In 1969, a serosurvey of nine common respiratory viruses, including rubella virus, was completed over a period of 3 months, among 112 children and adults of the Korekore tribe in northern Zimbabwe who visited a local mission hospital. Blood samples were tested for rubella antibodies using the haemagglutination inhibition assay. The study found 88% rubella seropositivity among the study population [12]. This high rate of seropositivity suggested a recent rubella outbreak around the time of the serosurvey. A measles and rubella epidemic occurred in Zimbabwe in 1977–1978, which resulted in sudden detection of an unexpected number of cases of rubella embryopathy in infants born at a major African referral hospital [13]. It was suggested that this epidemic was fuelled by population movement because of the escalation of war during the same period. In the literature, it has been suggested that rubella epidemics usually occur at intervals of approximately 7–10 years. This is also supported by data from The Gambia where epidemics were serologically diagnosed during 1963–1964 and 1973–1974 [14].

Owing to the existing burden of rubella in some regions of the world, the WHO recommends introduction of rubella-containing vaccines as a strategy to control the disease. Rubella vaccination programmes are economically justifiable and have demonstrated cost-effectiveness. Published economic analyses for the period between 1980 and 2010 indicate that the annual cost (inflation-adjusted 2012 US$) for the care of a patient with CRS in middle-income countries ranges from US$4,200 in Brazil to US$58,000 in Panama, whereas the lifetime cost for the care of a patient with CRS in high-income countries (inflation-adjusted 2012 US$) is estimated to be US$139,900 in Oman and over US$200,000 in the United States [15].

The WHO recommends that prior to introduction of rubella vaccine to national immunization programmes, rubella surveillance should be implemented through the existing measles surveillance system to estimate the disease burden [3]. In line with the WHO strategy for rubella vaccination in Africa, our analysis of data from Zimbabwe’s combined measles/rubella case-based surveillance system aims to describe the trends and population demographics of children and adolescents with rubella in the country, who were identified through its national surveillance system during a 5-year period between 2007 and 2011.

## Methods

### Sample collection and laboratory procedures

During the study period, blood samples were collected from suspected measles cases detected within 14 days of rash onset, according to the standard WHO/AFRO case definitions for measles and rubella surveillance. All age groups were investigated, including adults. According to the Zimbabwe Ministry of Health guidelines [16] and WHO guidelines for measles surveillance [10], all suspected cases should be tested for measles IgM antibodies. However, if an outbreak of measles is suspected, only the first five cases from a cluster of suspected measles cases should be tested for measles IgM antibodies. If three or more of these cases test IgM-positive, this is considered a laboratory-confirmed measles outbreak. When an outbreak is confirmed, no further samples should be collected from that district or area until 30 days after initial confirmation of the outbreak. Additional suspected cases of measles detected within 30 days in the same district or area are line listed as epidemiologically linked or clinically confirmed measles cases. A regional outbreak of measles in seven southern African countries including Zimbabwe was confirmed during 2009–2010 [17]. Sample collection and testing were therefore restricted during this time. Supplementary measles vaccination activities were subsequently carried out to contain this outbreak.

At first contact with a suspected case, about 1–5-ml blood was collected by venipuncture into a sterile anticoagulant-free tube. The blood was allowed to clot and then centrifuged at 3000 rpm for 5 minutes to separate the serum. If there was no centrifuge at the health centre, the blood specimen was kept in a refrigerator until there was complete retraction of the clot from the serum. The serum was transferred aseptically to a sterile vial, and then stored at 2–8C for no more than 3 days before being transported to the testing laboratory in cold boxes at the same temperature. All blood samples were tested at the National Virology Reference Laboratory (NVRL), which houses the National Measles Laboratory. The latter is accredited by the WHO Global Measles and Rubella Laboratory Network (LabNet) and thus generates credible results for the programme.

### Serological testing for rubella virus IgM antibodies

Serum samples were stored at −30°C (±2°C) and tested within 7 days. Enzyme-linked immunosorbent assay (ELISA) for rubella-specific IgM antibody was performed according to the manufacturer’s protocol (Enzygnost® Anti-Rubella Virus/IgM kit; Siemens AG, Erlangen, Germany). A micropipette (Gilson International B.V., Den Haag, the Netherlands) was used to manually dispense the samples and reagents. Twenty microlitres of serum were used in the test. Test plates were washed using a microplate washer (ELx50; BioTek UK, Bedfordshire, UK). Optical densities were read at 450 nm with a 630-nm reference filter using an ELISA reader (ELx808i; BioTek UK). All samples were tested once and those producing equivocal results were not retested owing to a lack of resources.

### Ethical issues

Zimbabwe’s combined measles/rubella surveillance is a national programme approved by the Ministry of Health and Child Care, which is supported by WHO/AFRO as part of the global goal to control and eliminate measles and rubella. All procedures for obtaining patient information and specimens were performed according to the WHO/AFRO measles/rubella surveillance protocol. Although written consent from suspected cases (or their parents, in the case of minor children) is not required for purposes of the national surveillance system, relevant information was provided and verbal permission sought before collection of samples.

## Results

**Figure 1:**
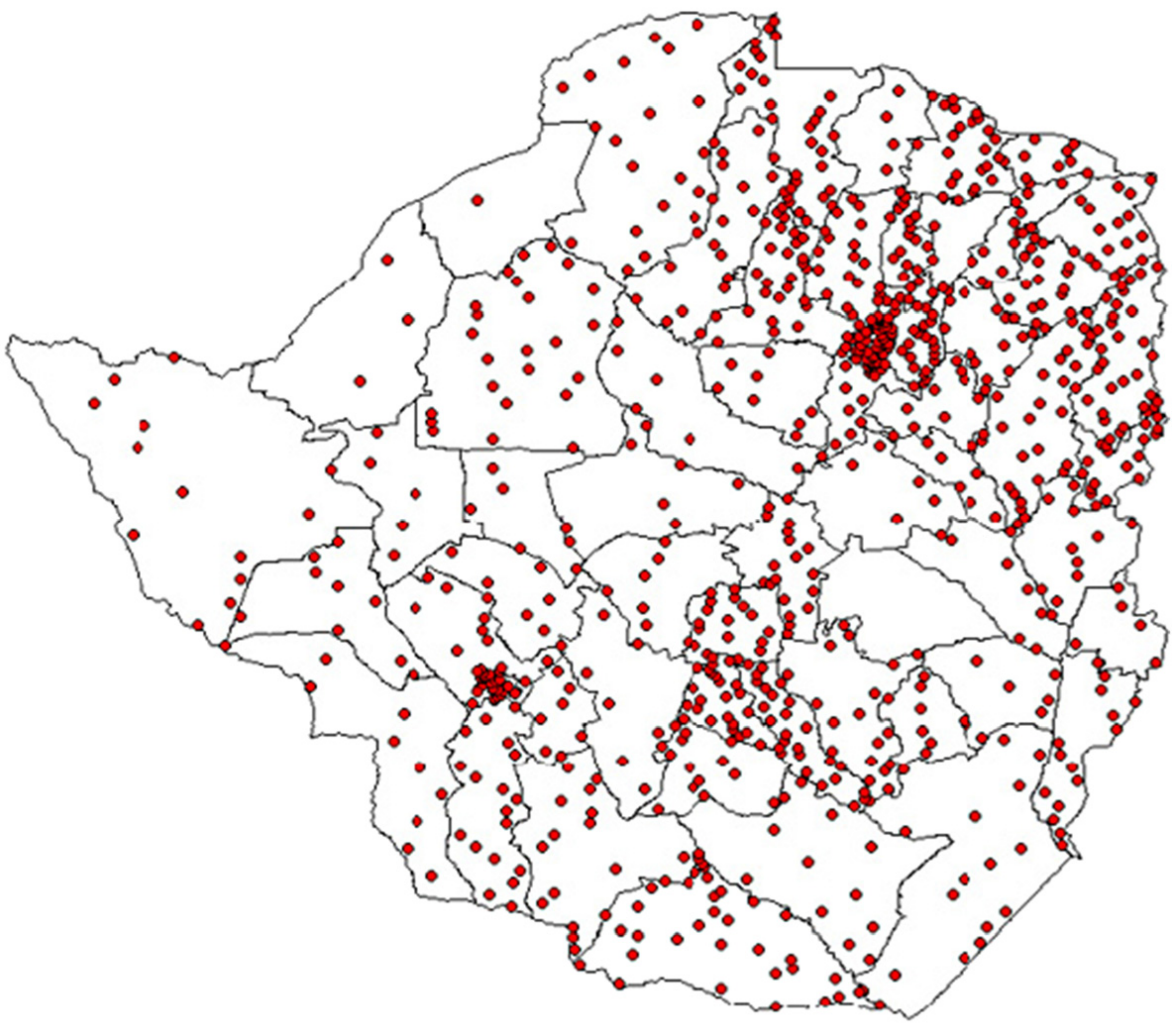
**Spot map of Zimbabwe showing the distribution of laboratory confirmed rubella cases (2007–2011).** The map of Zimbabwe showing district boundaries; one dot represents one confirmed case of rubella. This distribution shows that all districts were affected with tendency to cluster around urban areas owing to higher population densities in urban compared to rural areas.

During the 5 years under review, a total of 3428 samples from suspected measles cases were received by the National Measles Laboratory, from all districts and cities of Zimbabwe. Most cases originated from the city of Harare (655; 19.1%) and the adjacent district of Goromonzi (108; 3.2%). Other districts had less than 100 suspected cases during each year of the study period. The geographical distribution of these cases is shown in Figure 1. Of samples from suspected measles cases, 2999 (87.5%) were tested for measles IgM antibodies; 429 samples were not tested because they were collected within 30 days after confirmation of a regional measles outbreak during 2009–2010. Cases comprised 1757 (51.2%) males and 1671 (48.8%) females.

**Figure 2:**
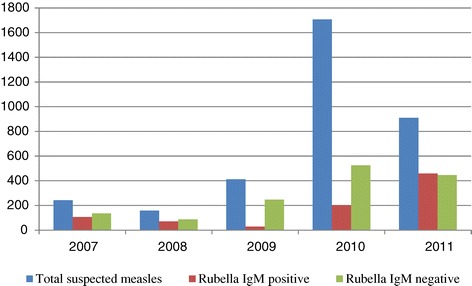
**Number of rubella IgM positive and negative samples among suspected measles cases, 2007–2011, Zimbabwe.** An outbreak of measles was confirmed in 2009 and supplementary immunization activities started. The outbreak was controlled by 2010. In 2011 the number samples from suspected measles cases referred to the laboratory for testing remained high. Fifty percent of these cases were positive for rubella.

Of the 3428 samples received by the laboratory, 2302 (67.2%) were eligible for rubella testing. A total of 865 (37.6%) were rubella IgM-positive, as shown in Table 1. During the measles outbreak in 2009–2010, laboratory detection of rubella IgM antibody was low. Although there was no confirmed case of measles after this outbreak, the number of samples from suspected measles cases received by the laboratory remained high (Figure 2). Of the 910 samples received in 2011, all were negative for measles antibodies whereas 50.7% tested positive for rubella IgM.

**Table 1:** **Distribution of suspected and confirmed measles and rubella cases, 2007–2011**

**YEAR**	**Total received for measles testing**	**Total tested for measles**	**Number (%) positive for measles IgM**	**Total tested for rubella**	**Number (%) positive for rubella IgM**
2007	242	242	1 (0.4)	241	106 (44)
2008	158	158	0 (0)	158	71 (44.9)
2009	412	400	125 (31.3)	275	29 (10.5)
2010	1706	1296	571 (44.1)	725	201 (27.7)
2011	910	903	0 (0)	903	458 (50.7)
Total	3428	2999	697 (23.2)	2302	865 (37.6)

The table shows a breakdown by year of number of samples from suspected measles cases identified through the national measles and rubella surveillance system during 2007–2011. Samples received at the National Measles Laboratory in good condition were tested first for measles and the numbers that were positive along with their percentages (%) are indicated. Samples that were negative for measles were subsequently tested for rubella and the total positives, together with their percentages (%) are also indicated. In 2007, for example, only 1 (0.4%) of 242 samples tested for measles at the laboratory was positive. Of 241 measles negative samples, 106 (44%) were positive for rubella.

**Figure 3:**
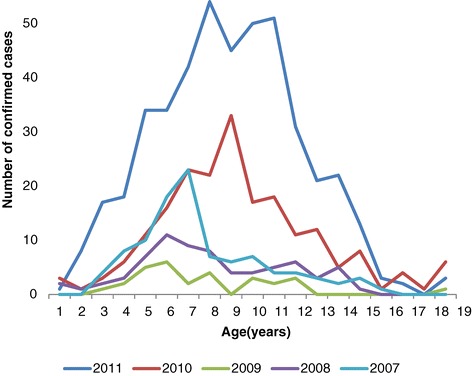
**Age incidence of laboratory confirmed rubella, 2007–2011, Zimbabwe.** The graph shows age incidence of rubella infection during each year. The incidence rises from 3 to 11 years of age and starts to decline. Cases older than 15 years were also detected by the surveillance. These represent susceptible build-up in older age groups of the female population following local extinction of rubella in the inter-epidemic periods.

The incidence of laboratory-confirmed rubella showed an increase in children over 3 years of age, followed by a decline after 12 years (Figure 3). Peak incidence occurred between 7 and 12 years, and 98% of confirmed cases were under 15 years old; age was not specified in fifteen cases (1.7%).

**Figure 4:**
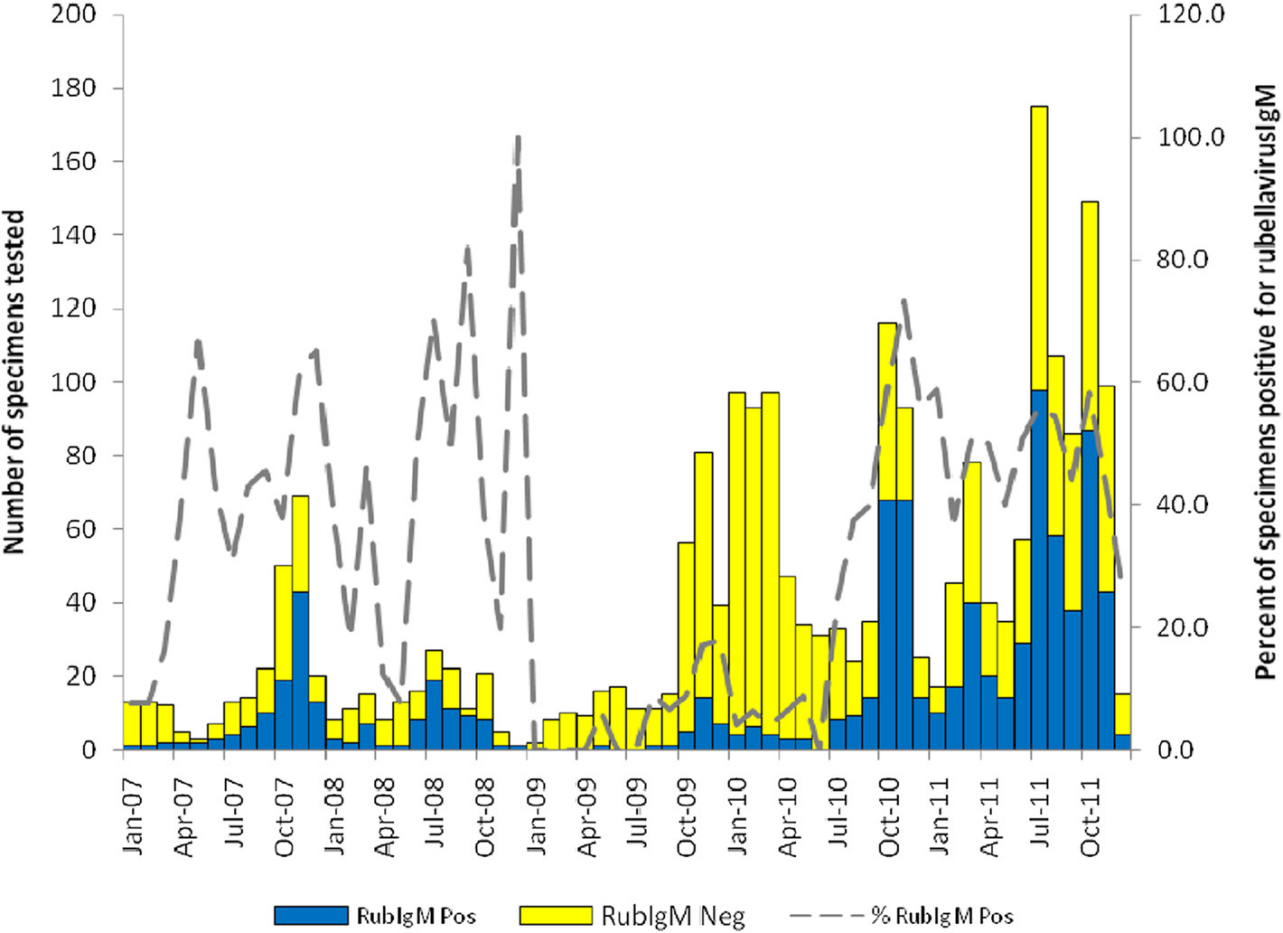
**Seasonal pattern of rubella infection in Zimbabwe.** The graph shows monthly incidence of laboratory confirmed rubella during a five year period. In each year larger peaks occurred during the dry months particularly late spring (October and November).

There was a seasonal pattern in the occurrence of laboratory confirmed rubella, with peaks in the late spring (October to November) as shown in Figure 4. There was a sharp decline in December and incidence remained low through April of each year. December is usually characterized by heavy rainfall and the rainy season lasts through April; May to mid-November is generally dry. Small peaks were observed in autumn (March to April). The mean incidence of laboratory confirmed rubella among the suspected cases was 37.5% (95% CI = 34.2-40.8) during rainy seasons and 62.5% (95% CI = 59.2-65.8) during dry seasons.

## Discussion

Concurrent measles and rubella surveillance enabled detection of rubella trends in Zimbabwe. In the current analysis, we observed that during the measles outbreak of 2009–2010, laboratory detection of rubella was low (10.5–27.7%) compared with the years 2007, 2008, and 2011, during which the incidence of laboratory-confirmed rubella was 44–50.7%. This observation could be a reflection of the restricted laboratory testing during a measles outbreak. It also confirms observations from other studies that when the prevalence of measles is low the incidence of true measles among suspected cases meeting the case definition is low. Under such conditions measles-like illness is more likely due to other causes such as rubella. It may also suggest that the effectiveness of using the measles clinical case definition to detect rubella is poorer during a measles outbreak. The latter explanation is supported by Helfand et al. who carried out a 3-month survey to compare accuracy of the measles case definition with laboratory diagnosis for measles during a 1996–1997 measles outbreak in one province of Zimbabwe [18]. In their survey, serum samples obtained from 105 children (aged 1 to17 years) who presented to health facilities with suspected measles were tested for both measles and rubella-specific IgM. Of the 96 children who met the clinical case definition for measles, 72% were positive for measles IgM and 23% positive for rubella IgM; 3% were positive for both viruses and 2% were negative for both. Although the survey did not include a measles-free period for comparison, the 23% rubella seropositivity rate is in agreement with the rates observed during the measles epidemic in our analysis.

In another survey in South Africa, Blackburn et al. also found that rash-like illnesses fitting the surveillance criteria for measles were far more likely to be rubella. In this survey, 106 of 220 (48.2%) samples submitted for laboratory confirmation of measles were rubella IgM-positive whereas only 12 (5.5%) were positive for measles IgM. Additionally, 28 (12.7%) and two of these samples were positive for human herpesvirus 6 and parvovirus, respectively [19]. This 48.2% seropositivity rate for rubella is also in keeping with rates observed in Zimbabwe when measles incidence rates were zero percent per year.

The WHO clinical case definition used for measles surveillance (fever plus maculopapular rash plus cough, coryza or conjunctivitis) has limited sensitivity for rubella [20,21]. The positive predictive value of the clinical case definition for measles is dependent upon disease incidence. As measles incidence increases, the case definition becomes better at predicting measles. It is also known that up to 50% of children with rubella infections do not present with rash [22]. Rubella infection may present without fever and children often have minimal or no constitutional symptoms at all. The rash may be difficult to detect in people with dark skin. Several other agents may also cause measles-like rash such as parvovirus B19, adenovirus, human herpesvirus, enterovirus, and streptococcal bacteria. Therefore, it is likely that a significant proportion of children with rubella may have been missed through the existing surveillance system because it is designed to detect measles and not rubella.

Owing to limitations of the WHO case definition for identifying cases of rubella, modified definitions have been adopted in other regions that have set rubella elimination targets, so as to enhance detection. The following definition was used by the Pan American Health Organization for rubella surveillance as the region moved toward an elimination target: a suspected case is one in which a health worker suspects rubella [23]. The European Centre for Disease Prevention and Control also adopted a modified definition, namely, any person with sudden onset of generalized maculopapular rash and at least one of the following: cervical adenopathy, suboccipital adenopathy, post-auricular adenopathy, arthralgia or arthritis [24]. As Zimbabwe and other African countries work toward introducing rubella-containing vaccines and plan elimination goals, a modified case definition will need to be considered so as to improve detection rates.

The data in the present study also show that although rubella infections occurred throughout each year, they were characterized by large peaks in the months from August to November, which coincide with the dry and hot months in Zimbabwe. Smaller peaks were observed in March, which is normally a hot and rainy period. Seasonal variations of rubella incidence were noted in the United States and temperate regions during the prevaccine era, with major peaks occurring in the spring. Factors that have been implicated in the incidence of rubella include age (mean age of highest transmission rates 3–11 years), critical community size (population size required for the infection to persist locally is approximately 1,000,000, although it could be less because of underreporting), seasonality (incidence is highest in the spring), birth rate (incidence is high in communities with high birth rates), heterogeneity of vaccination coverage [25,26], school term (transmission increases with aggregation of children in school and decreases during school vacation) [27]. Geographical location plays an uncertain role. In one study in Peru, for example, the incidence of CRS was related to distance from the coastal region of Lima but no differences were found between an urban versus a rural setting in Argentina, Brazil, Chile, Jamaica, Trinidad and Uruguay [28]. Gender is not a significant factor for transmission. In our analysis, the age group most affected was 3–15 years old and there was no sex predilection noted.

Adolescents and young reproductive-age women were also affected during our study period. This may represent susceptible build-up in older age groups of the female population following local extinction of rubella in the inter-epidemic periods. This is of concern because in the absence of a routine and sustained high-coverage vaccination programme against rubella, there is high risk of giving birth to babies with CRS among this population of women. Our surveillance did not routinely collect pregnancy related data on reproductive age women or provide follow up because it was primarily designed to identify measles (not rubella) in 6 months to 15 years old. Collection of samples at health facility level was triggered by a suspicion of measles. It is therefore not known whether some of the adolescent girls and reproductive age women older than 15 years of age were pregnant at the time of diagnosis. In order to obtain this information the measles case report form used in the surveillance needs to be modified to include a pregnancy test for women older than 15 years who present with measles-like febrile rash illness.

The apparent higher disease incidence in Zimbabwe’s capital, Harare, and its peri-urban communities is more likely a reflection of higher population density and the dynamics of disease reporting in urban areas compared to rural areas. Remote areas may experience unique reporting challenges such as poor transportation, poor accessibility to health facilities, lower levels of health worker awareness and different health-seeking behaviour of community residents.

## Conclusions

Rubella virus circulates in all districts of Zimbabwe, mainly affecting children below the age of 15 years. Most cases occur during the dry season. Further studies are needed to characterize the rubella genotypes circulating in Zimbabwe, with a view to determine endemicity and trends so as to introduce and enhance vaccination interventions.

## Additional file

For more detail to readers an additional excel dataset file associated with all analysis in this articles is provided (see Additional file 1).

## Additional file

Additional file 1:
**This is Excel file containing dataset associated with all analysis in this article.** All variables that identify patients have been removed from the file. Variables included in this dataset are; date of onset, date of specimen collection, sex, age in years and months, measles IgM result and rubella IgM result. A result key is also provided.

## Abbreviations

CRS: congenital rubella syndrome
ELISA: enzyme-linked immunosorbent assay
IgG: immunoglobulin G
IgM: immunoglobulin M
NVRL: National Virology Reference Laboratory
WHO/AFRO: World Health Organization Regional Office for Africa
μl: microlitre
nm: nanometre
°C: degree Celscius Degrees
